# Risk factors for unfavourable outcomes after shunt surgery in patients with idiopathic normal-pressure hydrocephalus

**DOI:** 10.1038/s41598-022-18209-5

**Published:** 2022-08-17

**Authors:** Erena Kobayashi, Shigenori Kanno, Nobuko Kawakami, Wataru Narita, Makoto Saito, Keiko Endo, Masaki Iwasaki, Tomohiro Kawaguchi, Shigeki Yamada, Kazunari Ishii, Hiroaki Kazui, Masakazu Miyajima, Masatsune Ishikawa, Etsuro Mori, Teiji Tominaga, Fumiaki Tanaka, Kyoko Suzuki

**Affiliations:** 1 Department of Behavioral Neurology and Cognitive Neuroscience, Tohoku University Graduate School of Medicine, 2-1, Seiryo-machi, Aoba-ku, Sendai, Miyagi 980-8575 Japan; 2 Department of Neurology and Stroke Medicine, Yokohama City University Graduate School of Medicine, Yokohama, Japan; 3 Department of Neurosurgery, Southern Tohoku General Hospital, Iwanuma, Japan; 4 Department of Rehabilitation, Tohoku University Hospital, Sendai, Japan; 5 Department of Neurosurgery, National Center Hospital, National Center of Neurology and Psychiatry, Kodaira, Japan; 6 Department of Neurosurgery, Kohnan Hospital, Sendai, Japan; 7 Department of Neurosurgery, Shiga University of Medical Science, Otsu, Japan; 8 Department of Radiology, Kindai University Faculty of Medicine, Osaka, Japan; 9 Department of Neuropsychiatry, Kochi Medical School, Kochi University, Kochi, Japan; 10 Department of Neurosurgery, Juntendo University Faculty of Medicine, Tokyo, Japan; 11 Department of Neurosurgery and Normal Pressure Hydrocephalus Center, Rakuwakai Otowa Hospital, Kyoto, Japan; 12 Department of Behavioral Neurology and Cognitive Neuropsychiatry, Osaka University United Graduate School of Child Development, Suita, Japan; 13 Department of Neurosurgery, Tohoku University Graduate School of Medicine, Sendai, Japan

**Keywords:** Dementia, Hydrocephalus, Movement disorders

## Abstract

A number of vascular risk factors (VRFs) have been reported to be associated with idiopathic normal-pressure hydrocephalus (iNPH), but it remains unclear whether these VRFs are related to patient outcomes after shunt surgery. Therefore, we investigated the risk factors for unfavourable outcomes after shunt surgery in iNPH patients using two samples from Tohoku University Hospital and from a multicentre prospective trial of lumboperitoneal (LP) shunt surgery for patients with iNPH (SINPHONI-2). We enrolled 158 iNPH patients. We compared the prevalence of VRFs and clinical measures between patients with favourable and unfavourable outcomes and identified predictors of unfavourable outcomes using multivariate logistic regression analyses. The presence of hypertension, longer disease duration, more severe urinary dysfunction, and a lower Evans’ index were predictors of unfavourable outcomes after shunt surgery. In addition, hypertension and longer disease duration were also predictors in patients with independent walking, and a lower Evans’ index was the only predictor in patients who needed assistance to walk or could not walk. Our findings indicate that hypertension is the only VRF related to unfavourable outcomes after shunt surgery in iNPH patients. Larger-scale studies are needed to elucidate the reason why hypertension can affect the irreversibility of symptoms after shunt placement.

## Introduction

Normal-pressure hydrocephalus (NPH) is a syndrome associated with dilated ventricles and is characterized by gait disturbance, cognitive impairment, and urinary dysfunction^1^. NPH is divided into two groups, i.e., idiopathic NPH (iNPH), which has an insidious onset of unknown cause, and secondary NPH, which is caused by a disease or event such as subarachnoid haemorrhage, meningitis, cerebral contusion, or brain tumour. The early and accurate diagnosis of iNPH is becoming increasingly important because iNPH has a high prevalence in the elderly population and has been shown to be treatable with cerebrospinal fluid (CSF) shunt surgery^2–4^. However, older age is associated with a higher rate of comorbidities, which makes surgery and perioperative management more complicated. Therefore, we need to carefully determine the surgical indications for patients with iNPH not only to reduce the frequency of complications but also to ensure that we perform shunt surgery only in patients who are expected to benefit from it.

Hypertension is one of the most common risk factors for iNPH^5–7^. Other vascular risk factors (VRFs), such as diabetes mellitus and hyperlipidaemia, have also been reported to be associated with iNPH^8^. Although the actual pathophysiology of iNPH remains unclear, these studies indicate that an alteration in CSF dynamics due to vascular dysfunction may be related to the pathogenesis of iNPH. On the other hand, in the small number of studies that have reported on whether VRFs are associated with outcomes after shunt surgery^6,9–11^, the results have been inconsistent. Moreover, hypertension and diabetes mellitus have been reported to play integral roles not only in cerebrovascular diseases (CVDs) but also in the pathogenesis of Alzheimer’s disease (AD)^12–14^. Previous studies performing cortical biopsy for patients with iNPH have reported that AD pathology was present in 25–67.6% of patients with iNPH and was related to worse baseline cognitive performance and diminished postoperative outcomes in patients with iNPH^15–17^. Whether VRFs are actually related to the pathogenesis of iNPH should be reconsidered.

The primary purpose of this study was to investigate risk factors for unfavourable outcomes after shunt surgery and whether VRFs are associated with shunt responsiveness in patients with iNPH. The second purpose was to examine whether these risk factors are different between patients with relatively milder and more severe gait disturbance. In the early and middle stages of AD, abnormal gait has rarely been observed^18^. It is assumed that there would be a group of iNPH patients with more severe cognitive impairment relative to their walking performance due to a combination of AD and iNPH pathologies. Moreover, these patients would have poor postoperative outcomes because the AD pathology potentially contributes to the irreversibility of cognitive symptoms after shunt surgery^15,17,19^. If VRFs are more closely related to AD pathology than to iNPH pathology, relatively more severe cognitive impairments at baseline and unfavourable postoperative outcomes would be correlated with a high prevalence of VRFs in iNPH patients with relatively milder gait disturbances. We believe that it is important to investigate those associations to improve the postoperative outcomes of shunt surgery in patients with iNPH and to consider the diversity of its neuropathology. For these purposes, we analysed two samples, consisting of participants from Tohoku University Hospital and from a multicentre prospective trial of lumboperitoneal (LP) shunt surgery for patients with iNPH (SINPHONI-2)^4^.

## Methods

This study was conducted in accordance with the Declaration of Helsinki and approved by the Ethics Committee of Tohoku University Hospital (approval numbers: 2006-195, 2010-505, and 2020-1-285). Written informed consent was obtained from the patients and their families on the first admission.

SINPHONI-2 was conducted following the Guidelines for Good Clinical Practice and the Declaration of Helsinki. An independent committee monitored all the clinical data, imaging data, data related to safety issues, and protocol compliance through a web-based case report system. The study protocol was approved by the institutional review board at each site (see the SINPHONI-2 study centres below), and all patients or their representatives (if the patients could not understand the contents of the study) provided written informed consent before entry into the study.

### Participants from Tohoku University Hospital

One hundred forty-seven (61 women/86 men) consecutive patients with iNPH who were admitted to Tohoku University Hospital were enrolled between December 2006 and November 2016. All patients fulfilled the following criteria and underwent shunt surgery regardless of the response to the CSF tap test: (1) > 60 years of age; (2) gait disturbance, cognitive impairment, and/or urinary disturbance; (3) both ventricular dilation (Evans’ index > 0.3) and high convexity and medial subarachnoid space tightness on coronal MRI; (4) CSF pressure < 200 mmH_2_O with normal CSF cell counts and protein levels; (5) lack of a previous history of conditions that might cause ventricular dilation; and (6) the absence of other diseases that may account for such symptoms.

The choice of operative procedure, i.e., ventriculoperitoneal (VP) or LP shunt implantation, was determined according to the patient’s wishes and condition, such as the presence of spinal canal stenosis. The operations were conducted at Tohoku University Hospital or Kohnan Hospital. VP shunt implantation was conducted using a Codman-Hakim programmable valve with a Siphon-Guard (Codman and Shurtleff, Integra LifeSciences Corporation, Plainsboro, NJ, USA), and LP shunt implantation was conducted using a Codman-Hakim programmable valve with a Siphon-Guard or a Strata NSC adjustable-pressure valve with a Siphon-Control Device (Medtronic Inc., Minneapolis, MN, USA). Postoperatively, if clinical improvement was insufficient, pressure adjustments were made repeatedly until the optimal pressure for the patient was attained.

**Figure 1:**
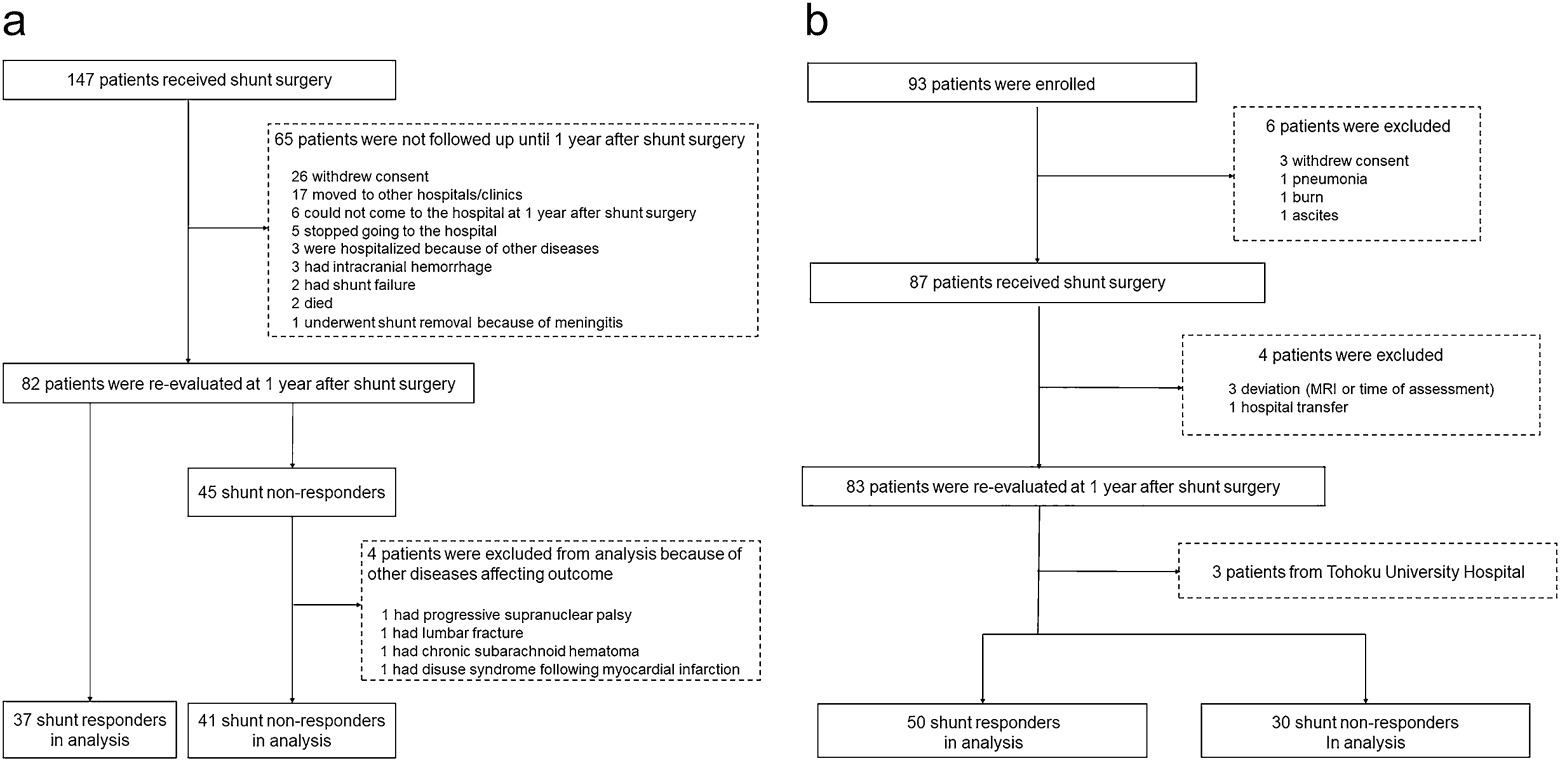
Patient flow charts in Tohoku University Hospital (**a**) and a multicentre prospective trial of lumboperitoneal (LP) shunt surgery for patients with iNPH (SINPHONI-2) (**b**).

Figure 1-a shows the flow chart of patient selection. Eighty-two of the initial set of patients were re-evaluated approximately 1 year (12 ± 1 months) after shunt surgery. The remaining 65 patients were not reassessed for various reasons. Moreover, four of the re-evaluated patients were excluded from the analysis because of complications that interfered with the postoperative evaluation of iNPH symptoms. Consequently, 78 patients were included in the following analyses.

### SINPHONI-2 study

Ninety-three (43 women/50 men) patients with iNPH were enrolled from 20 neurological and neurosurgical centres in Japan between March 2010 and October 2011. The inclusion criteria were (1) age between 60 and 85 years; (2) gait disturbance, cognitive impairment, and/or urinary disturbance; (3) both ventricular dilation (Evans’ index > 0.3) and high-convexity and medial subarachnoid space tightness on coronal MRI; (4) CSF pressure < 200 mmH_2_O with normal CSF cell counts and protein levels; (5) lack of a previous history of conditions that might cause ventricular dilation; (6) the absence of other diseases that may account for such symptoms; (7) a normal Queckenstedt’s test; and (8) the absence of severe vertebral degenerative diseases or spinal canal stenosis as shown on plain radiographs or MRI. Figure 1-b shows the flow chart for patient selection. Forty-nine (29 women/20 men) of the initial patients were randomly assigned to undergo LP shunt implantation within 1 month of randomization, and the remaining 44 (14 women/30 men) patients were assigned to delayed LP shunt implantation 3 months after randomization. All participants in both SINPHONI-2 groups underwent a tap test after randomization and were followed up until 12 months after shunt surgery. Consequently, 80 patients in both groups, except for three patients recruited from Tohoku University Hospital, were included in the following analyses. The details of the study were previously described^4^.

### Clinical assessments

In both studies, the patients underwent clinical assessment at baseline prior to both CSF removal and shunt implantation and were reassessed approximately 1 year after shunt implantation. We used the modified Rankin Scale (mRS) to define shunt responsiveness^20^. The mRS has been commonly used for evaluating physical disability or dependence in activities of daily living in acute stroke trials and was adopted for evaluating outcomes of shunt surgery in multicentre studies of iNPH^3,4^. In the current study, a favourable outcome of shunt implantation was defined as an improvement by one or more points on the mRS at the 1-year follow-up after shunt surgery. The severity of the triad was assessed using the idiopathic normal-pressure hydrocephalus grading scale (iNPHGS)^21^, and the patients were classified into the following two groups using the iNPHGS gait subscore: (1) the preserved walking performance group (iNPHGS gait subscore ≤ 2, i.e., patients who could walk independently) and (2) the reduced walking performance group (iNPHGS gait subscore ≥ 3, i.e., patients who needed assistance to walk or who could not walk).

We administered the timed up-and-go test (TUG) to assess gait function^22^. The completion time was used as the score. Patients who could not rise from an armchair and/or walk safely and independently were not assessed. Moreover, we administered the following neuropsychological measures: (1) the Mini-Mental State Examination (MMSE), whose total score was used to assess general cognitive function^23^; (2) the Frontal Assessment Battery (FAB), whose total score was used to assess executive function^24^; and (3) the Trail Making test A (TMT-A), whose completion time was used to assess psychomotor speed^25^;if a patient could not complete the task within the time limit of 420 s, the patient was given a score of 420 s.

### Vascular risk factors

We investigated the VRFs in each patient. The criteria for these factors were as follows: (1) hypertension: systolic blood pressure ≥ 140 and/or diastolic blood pressure ≥ 90 mmHg, or use of antihypertensive drugs; (2) diabetes mellitus: fasting plasma glucose level ≥ 126 mg/dl and haemoglobin A1c level ≥ 6.5% or usage of hypoglycaemic drugs; (3) hyperlipidaemia; plasma low-density lipoproteins ≥ 140 mg/dl and/or triglycerides ≥ 150 mg/dl or use of a lipid-lowering drug; (4) smoking: current or past smoking; and (5) ischaemic stroke: a history of cerebral infarction (lacunar stroke, atherothrombotic stroke, cardiogenic embolic stroke, and/or haemodynamic stroke) or transient ischaemic attack or the presence of ischaemic lesions on presurgical MRI. We prospectively checked these data except for the presence of ischaemic lesions on presurgical MRI (retrospectively). In the present study, periventricular hyperintensity and deep white matter hyperintensity were not assessed to avoid overdetection of ischaemic lesions.

### Statistical analyses

The demographic data, the prevalence of VRFs, and clinical measures at baseline were compared in all participants and in each walking performance group as follows: (1) the participants from Tohoku University Hospital versus SINPHONI-2; and (2) the patients with favourable versus unfavourable outcomes. Furthermore, to discuss the differences in the results between our study and Nakajima et al.’s previous studies, we compared the prevalence of each VRF that was significantly different between the patients with favourable and unfavourable outcomes among patients with the same mRS score (grade 1–2, grade 3, or grade 4)^9^.

We used the chi-square test to compare the sex distribution, the prevalence of VRFs, the operative procedure, and shunt responsiveness between groups. Student’s t test was used for age, educational attainment, disease duration, Evans’ index, and the scores of neuropsychological and gait tests. The Mann–Whitney U test was used for the mRS score and the iNPHGS total and subscores. To evaluate the changes in clinical measures after shunt implantation among only patients with favourable or unfavourable outcomes, the paired Student’s t test was used to compare the scores of neuropsychological and gait tests, and the Wilcoxon signed-ranked test was used to compare the mRS score and the iNPHGS total and subscores.

Finally, we performed multivariate logistic regression analysis by the forced-entry method to identify the predictors of unfavourable outcome in all participants and in each walking performance group. Each independent variable included in these analyses was selected when its p value for comparisons between patients with favourable and unfavourable outcomes was below 0.1. Multicollinearity among independent variables was identified based on the variance inflation factor (VIF).

Statistical analyses were performed with SPSS statistics for version 25.0 (IBM, Armonk, New York). A p value < 0.05 was considered statistically significant.

## Results

### Demographic and clinical characteristics of participants at baseline

The demographic data and the results of clinical measures at baseline are shown in Table 1. We failed to investigate educational attainment in 12 patients and disease duration in one patient. We did not use the Evans’ index of one patient because of the presence of cavum vergae. In addition, we lost the Evans’ index of one patient from the SINPHONI-2 trial due to data collection failure. Two patients did not complete the FAB, and 15 patients did not complete the TMT-A because of severe cognitive impairments or refusal. Ten patients did not undergo the TUG because they could not rise from the armchair and/or walk safely and independently. Although we could check the presence of ischaemic lesions on presurgical MRI in all patients from Tohoku University Hospital, we could not check this in 23 patients from SINPHONI-2 retrospectively.

**Table 1:** Demographics, clinical measures, and prevalence of vascular risk factors at baseline.

Variable	All samples	Tohoku University Hospital	SINPHONI-2	p value
**Number**				
All participants	158	78	80	
Preserved WP group	99	57	42	
Reduced WP group	59	21	38	
**Age (years), mean ± SD**				
All participants	76.4 ± 4.6	76.4 ± 4.5	76.3 ± 5.0	0.935^†^
Preserved WP group	76.0 ± 4.7	76.6 ± 4.5	75.3 ± 5.0	0.204^†^
Reduced WP group	77.0 ± 4.3	76.0 ± 4.5	77.5 ± 4.2	0.216^†^
**Sex (women/men)**				
All participants	75/83	39/39	36/44	0.529^‡^
Preserved WP group	42/57	24/33	18/24	0.940^‡^
Reduced WP group	33/26	15/6	18/20	0.075^‡^
**Educational attainment (years), mean ± SD**				
All participants	11.8 ± 3.2 (n = 146)	11.1 ± 2.9	12.5 ± 3.5 (n = 68)	0.010^†^
Preserved WP group	11.7 ± 3.3 (n = 90)	11.2 ± 3.1	12.5 ± 3.3 (n = 33)	0.070^†^
Reduced WP group	11.9 ± 3.2 (n = 56)	10.9 ± 2.0	12.5 ± 3.7 (n = 35)	0.039^†^
**Disease duration (years), mean ± SD**				
All participants	2.9 ± 2.3 (n = 156)	3.6 ± 2.4 (n = 77)	2.1 ± 1.9 (n = 79)	< 0.001^†^
Preserved WP group	2.9 ± 2.2 (n = 97)	3.7 ± 2.3 (n = 56)	1.9 ± 1.4 (n = 41)	< 0.001^†^
Reduced WP group	2.7 ± 2.4	3.4 ± 2.6	2.4 ± 2.3	0.112^†^
**Evans’ index, mean ± SD**				
All participants	0.344 ± 0.028 (n = 156)	0.351 ± 0.028 (n = 77)	0.336 ± 0.027 (n = 79)	< 0.001^†^
Preserved WP group	0.345 ± 0.028 (n = 97)	0.353 ± 0.027 (n = 56)	0.335 ± 0.025 (n = 41)	0.001^†^
Reduced WP group	0.342 ± 0.029	0.348 ± 0.030	0.338 ± 0.028	0.200^†^
**mRS, median (25th–75th percentile)**				
All participants	3.0 (2.0–3.0)	3.0 (2.0–3.0)	3.0 (2.0–4.0)	0.042^§^
Preserved WP group	2.0 (2.0–3.0)	2.0 (2.0–3.0)	2.0 (2.0–3.0)	0.903^§^
Reduced WP group	4.0 (3.0–4.0)	3.0 (3.0–4.0)	4.0 (3.0–4.0)	0.631^§^
**iNPHGS, median (25th–75th percentile)**				
Gait				
All participants	2.0 (2.0–3.0)	2.0 (2.0–3.0)	2.0 (2.0–3.0)	0.061^§^
Preserved WP group	2.0 (2.0–2.0)	2.0 (2.0–2.0)	2.0 (2.0–2.0)	0.077^§^
Reduced WP group	3.0 (3.0–3.0)	3.0 (3.0–3.0)	3.0 (3.0–3.0)	0.934^§^
Cognition				
All participants	2.0 (2.0–3.0)	2.0 (2.0–3.0)	2.0 (2.0–3.0)	0.955^§^
Preserved WP group	2.0 (2.0–3.0)	2.0 (2.0–3.0)	2.0 (1.0–3.0)	0.425^§^
Reduced WP group	3.0 (2.0–3.0)	3.0 (2.0–3.0)	3.0 (2.0–3.0)	0.835^§^
Urination				
All participants	2.0 (1.0–3.0)	1.5 (1.0–3.0)	2.0 (1.0–3.0)	0.160^§^
Preserved WP group	1.0 (1.0–2.0)	1.0 (1.0–2.0)	2.0 (1.0–2.0)	0.556^§^
Reduced WP group	2.0 (1.0–3.0)	2.0 (1.0–3.0)	2.0 (2.0–3.0)	0.575^§^
Total				
All participants	6.0 (5.0–8.0)	6.0 (5.0–7.0)	6.5 (5.0–6.0)	0.176^§^
Preserved WP group	6.0 (4.0–7.0)	6.0 (5.0–7.0)	6.0 (4.0–6.0)	0.564^§^
Reduced WP group	8.0 (7.0–9.0)	8.0 (6.5–9.0)	8.0 (7.0–9.0)	0.610^§^
MMSE (/30), mean ± SD				
All participants	20.9 ± 5.8	22.2 ± 4.5	19.7 ± 6.6	0.006^†^
Preserved WP group	22.4 ± 4.7	22.7 ± 4.1	22.0 ± 5.4	0.437^†^
Reduced WP group	18.4 ± 6.5	20.6 ± 5.2	17.2 ± 6.8	0.048^†^
FAB (/18), mean ± SD				
All participants	9.8 ± 3.6 (n = 156)	10.3 ± 3.1	9.3 ± 4.0 (n = 78)	0.087^†^
Preserved WP group	10.7 ± 3.2 (n = 98)	10.7 ± 2.8	10.7 ± 3.6 (n = 41)	0.978^†^
Reduced WP group	8.3 ± 3.8 (n = 58)	9.1 ± 3.6	7.8 ± 3.8 (n = 37)	0.183^†^
TMT-A (s), mean ± SD				
All participants	143 ± 111 (n = 143)	141 ± 112	145 ± 111 (n = 65)	0.856^†^
Preserved WP group	118 ± 89 (n = 94)	119 ± 93	115 ± 83 (n = 37)	0.832^†^
Reduced WP group	191 ± 133 (n = 49)	200 ± 137	183 ± 132 (n = 28)	0.661^†^
TUG (s), mean ± SD				
All participants	24.1 ± 30.3 (n = 148)	17.1 ± 23.9 (n = 69)	30.2 ± 33.9 (n = 79)	0.007^†^
Preserved WP group	14.3 ± 4.8	13.1 ± 3.8	15.9 ± 5.6	0.004^†^
Reduced WP group	44.0 ± 46.4 (n = 49)	36.2 ± 54.5 (n = 12)	46.5 ± 44.0 (n = 37)	0.508^†^
Hypertension				
All participants	90 (57.0%)	46 (59.0%)	44 (55.0%)	0.616^‡^
Preserved WP group	59 (59.6%)	34 (59.6%)	25 (59.5%)	0.990^‡^
Reduced WP group	31 (52.5%)	12 (57.1%)	19 (50.0%)	0.599^‡^
Diabetes mellitus				
All participants	40 (25.3%)	21 (26.9%)	19 (23.8%)	0.647^‡^
Preserved WP group	27 (27.3%)	17 (29.8%)	10 (23.8%)	0.507^‡^
Reduced WP group	13 (22.0%)	4 (19.0%)	9 (23.7%)	0.681^‡^
Hyperlipidaemia				
All participants	65 (41.1%)	39 (50.0%)	26 (32.5%)	0.025^‡^
Preserved WP group	44 (44.4%)	29 (50.9%)	15 (35.7%)	0.133^‡^
Reduced WP group	21 (35.6%)	10 (47.6%)	11 (28.9%)	0.152^‡^
Smoking				
All participants	47 (29.7%)	26 (33.3%)	21 (26.3%)	0.330^‡^
Preserved WP group	35 (35.4%)	22 (38.6%)	13 (31.0%)	0.432^‡^
Reduced WP group	12 (20.3%)	4 (19.0%)	13 (52%) (n = 25)	0.536^‡^
Ischaemic stroke				
All participants	64 (47.4%) (n = 135)	37 (47.4%)	27 (47.4%) (n = 57)	0.994^‡^
Preserved WP group	42 (47.2%) (n = 89)	28 (49.1%)	14 (43.8%) (n = 32)	0.626^‡^
Reduced WP group	22 (47.8%) (n = 46)	9 (42.9%)	13 (52%) (n = 25)	0.536^‡^
Shunt procedures (VP/LP)				
All participants	51/107	51/27	0/80	< 0.001^‡^
Preserved WP group	34/65	34/23	0/42	< 0.001^‡^
Reduced WP group	17/42	17/4	0/38	< 0.001^‡^
Outcomes (favourable/unfavourable)				
All participants	87 (55.1%)/71	37 (47.4%)/41	50 (62.5%)/30	0.057^‡^
Preserved WP group	51 (51.5%)/48	26 (45.6%)/31	25 (59.5%)/17	0.171^‡^
Reduced WP group	36 (61.0%)/23	11 (52.4%)/10	25 (65.8%)/13	0.312^‡^

*FAB* Frontal Assessment Battery, *iNPH* idiopathic normal-pressure hydrocephalus, *iNPHGS* idiopathic Normal-Pressure Hydrocephalus Grading Scale, *LP* lumboperitoneal, *MMSE* Mini-Mental State Examination, *TMT-A* Trail Making Test A, *SINPHONI-2* a multicentre prospective trial of lumboperitoneal shunt surgery for patients with idiopathic normal-pressure hydrocephalus, *TUG* timed up-and-go test, *VP* ventriculoperitoneal, *WP* walking performance.

^†^The Student’s t-test was used.

^‡^The chi-square was used.

^§^The Mann–Whitney U-test was used.

The analyses targeting all participants demonstrated that the participants from Tohoku University Hospital had a significantly longer disease duration, lower educational attainment, greater Evans’ index, lower mRS scores, shorter TUG completion time, higher MMSE total scores, and a higher prevalence of hyperlipidaemia than those from SINPHONI-2. In addition, the participants from Tohoku University Hospital tended to show lower iNPHGS gait subscores, lower total FAB scores, and poorer shunt response than those from SINPHONI-2. There was no apparent difference in age, sex ratio, iNPHGS cognition or urination subscore or total score, TMT-A score, or the prevalence of any VRF except for hyperlipidaemia between the participants from Tohoku University Hospital and from SINPHONI-2.

In the preserved walking performance group, the participants from Tohoku University Hospital had a significantly longer disease duration, greater Evans’ index, and shorter TUG completion time than those from SINPHONI-2. The participants from Tohoku University Hospital also tended to show higher iNPHGS gait subscores and lower educational attainment than those from SINPHONI-2. There was no apparent difference in age, sex ratio, mRS score, iNPHGS cognition or urination subscore or total score, the score of any neuropsychological test we administered, or the prevalence of any VRF between the preserved walking performance participants from Tohoku University Hospital and SINPHONI-2.

In the reduced walking performance group, the participants from Tohoku University Hospital had significantly lower educational attainment and higher MMSE total scores than those from SINPHONI-2. In addition, there tended to be more female participants from Tohoku University Hospital than from SINPHONI-2. There was no apparent difference in age, Evans’ index, mRS score, any iNPHGS subscore or total score, FAB score, TMT-A score, TUG completion time, or the prevalence of any VRF between the reduced walking performance participants from Tohoku University Hospital and SINPHONI-2.

Although there was no significant difference in shunt surgery outcomes between the participants from each walking performance group, the proportion of patients with favourable outcomes from Tohoku University Hospital tended to be lower than that from SINPHONI-2 (Tohoku University Hospital: 47.4% (37/78), SINPHONI-2: 62.5% (50/80), p = 0.057). Supplementary Table 1 lists the adverse events during the 1-year follow-up period at Tohoku University Hospital. These patients had fewer serious adverse events (defined as those resulting in hospitalization, prolongation of hospitalization, and significant disability) than the participants from SINPHONI-2^4^.

### Clinical measures and the VRFs associated with shunt responsiveness

**Figure 2:**
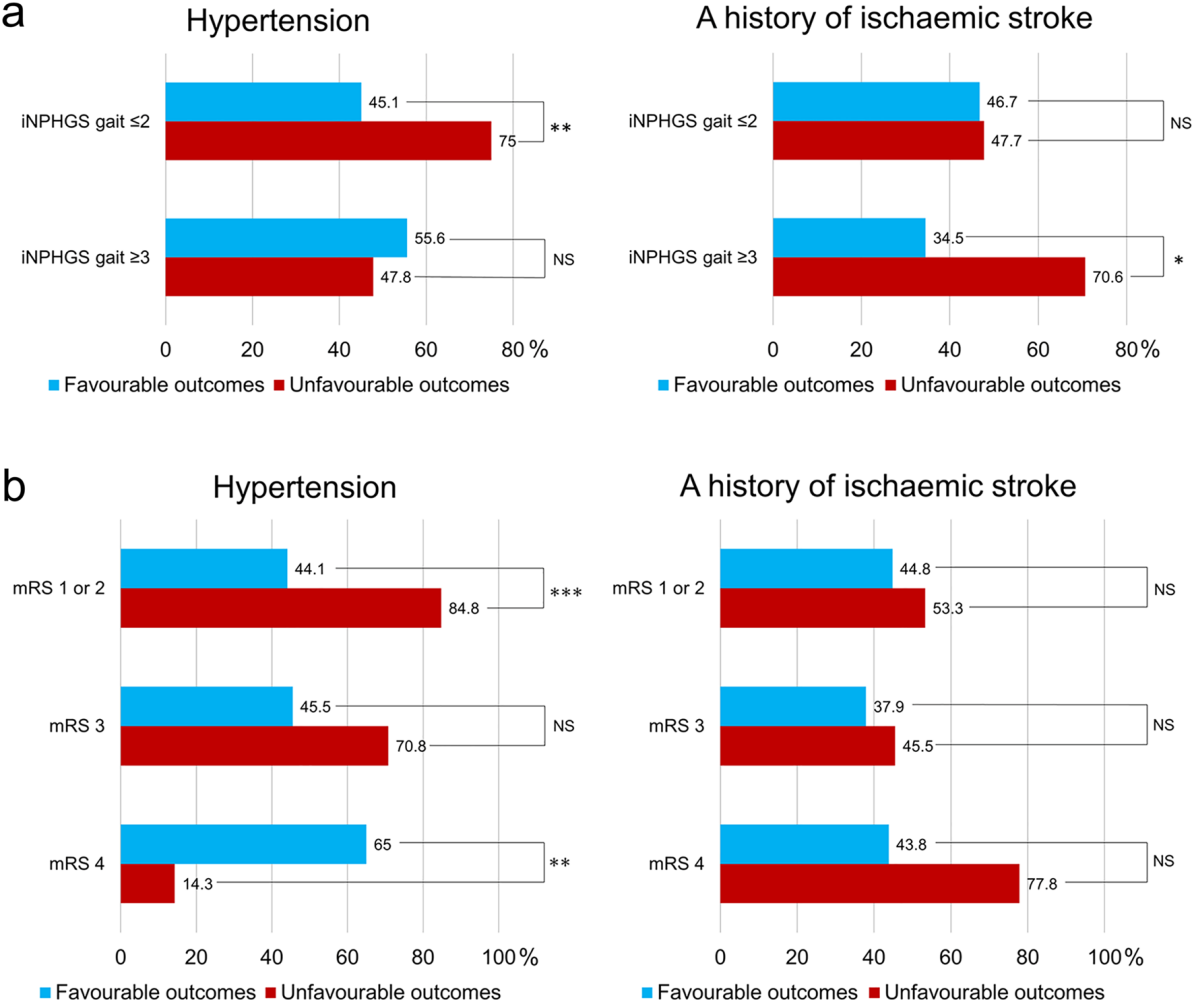
The prevalence of hypertension and ischaemic stroke history. (**a**) Patients with iNPHGS gait subscores of 2 or below and with iNPHGS gait subscores of 3 or above. (**b**) Patients with an mRS grade 1 or 2, grade 3, and grade 4. *iNPHGS* idiopathic normal-pressure hydrocephalus grading scale, *mRS* modified Rankin Scale. *p < 0.05, **p < 0.005, ***p < 0.001.

The differences in demographics and clinical measures between the patients with favourable and unfavourable outcomes are shown in Table 2. The analyses targeting all participants demonstrated that the participants with unfavourable outcomes showed a significantly longer disease duration, lower Evans’ index, and a higher prevalence of hypertension than those with favourable outcomes. In addition, the median iNPHGS urination subscore of patients with unfavourable outcomes tended to be higher than that of patients with favourable outcomes. In the preserved walking performance group, the prevalence of hypertension in patients with unfavourable outcomes was significantly higher than in those with favourable outcomes (Fig. 2-a). In addition, patients with unfavourable outcomes tended to show a longer disease duration, lower total MMSE and FAB scores, and a higher prevalence of diabetes mellitus than those with favourable outcomes. In the reduced walking performance group, patients with unfavourable outcomes showed a significantly lower Evans’ index and a higher prevalence of a history of ischaemic stroke than those with favourable outcomes (Fig. 2-a). Moreover, the median iNPHGS urination subscore of patients with unfavourable outcomes tended to be higher than that of patients with favourable outcomes.

**Table 2:** Clinical differences between the patients with favourable and unfavourable outcomes.

Variable	Patients with favourable outcomes	Patients with unfavourable outcomes	p value
**Number (Tohoku University Hospital/SINPHONI-2)**			
All participants	37/50	41/30	0.057^†^
Preserved WP group	26/25	31/17	0.171^†^
Reduced WP group	11/25	10/13	0.312^†^
**Age (years), mean ± SD**			
All participants	76.3 ± 4.7	76.4 ± 4.5	0.916^‡^
Preserved WP group	76.1 ± 5.1	76.0 ± 4.4	0.935
Reduced WP group	76.7 ± 4.1	77.3 ± 4.8	0.620^‡‡^
**Sex (women/men)**			
All participants	44/43	31/40	0.387^†^
Preserved WP group	23/28	19/29	0.579^†^
Reduced WP group	21/15	12/11	0.642^†^
**Educational attainment (years), mean ± SD**			
All participants	12.0 ± 3.5 (n = 78)	11.5 ± 2.9 (n = 68)	0.454^‡^
Preserved WP group	12.0 ± 3.6 (n = 45)	11.3 ± 2.8 (n = 45)	0.274^‡^
Reduced WP group	11.8 ± 3.5 (n = 33)	12.0 ± 3.0	0.801^‡^
**Disease duration (years), mean ± SD**			
All participants	2.5 ± 1.8 (n = 86)	3.3 ± 2.7 (n = 70)	0.047^‡^
Preserved WP group	2.6 ± 1.8 (n = 50)	3.3 ± 2.5 (n = 47)	0.075^‡^
Reduced WP group	2.5 ± 1.9	3.1 ± 3.1	0.314^‡^
**Evans’ index, mean ± SD**			
All participants	0.349 ± 0.031	0.337 ± 0.023 (n = 69)	0.006^‡^
Preserved WP group	0.348 ± 0.032	0.342 ± 0.022 (n = 46)	0.262^‡^
Reduced WP group	0.351 ± 0.030	0.327 ± 0.022	0.002^‡^
**mRS, median (25th–75th percentile)**			
All participants	3.0 (2.0–3.0)	3.0 (2.0–3.0)	0.255^§^
Preserved WP group	2.0 (2.0–3.0)	2.0 (2.0–3.0)	0.181^§^
Reduced WP group	3.0 (3.0–4.0)	4.0 (3.0–4.0)	0.212^§^
**iNPHGS, median (25th–75th percentile)**			
Gait			
All participants	2.0 (2.0–3.0)	2.0 (2.0–3.0)	0.303^§^
Preserved WP group	2.0 (2.0–2.0)	2.0 (2.0–2.0)	0.895^§^
Reduced WP group	3.0 (3.0–3.0)	3.0 (3.0–3.0)	0.317^§^
Cognition			
All participants	2.0 (3.0–3.0)	2.0 (2.0–3.0)	0.816^§^
Preserved WP group	2.0 (1.0–3.0)	2.0 (2.0–3.0)	0.545^§^
Reduced WP group	3.0 (2.0–3.0)	3.0 (2.0–3.0)	0.565^§^
Urination			
All participants	2.0 (1.0–2.0)	2.0 (1.0–3.0)	0.094^§^
Preserved WP group	1.0 (1.0–2.0)	1.5 (1.0–3.0)	0.286^§^
Reduced WP group	2.0 (1.0–3.0)	3.0 (2.0–3.0)	0.062^§^
Total			
All participants	6.0 (5.0–6.0)	6.0 (5.0–8.0)	0.601^§^
Preserved WP group	5.0 (4.0–6.0)	6.0 (5.0–7.0)	0.214^§^
Reduced WP group	8.0 (7.0–9.0)	9.0 (6.0–9.0)	0.174^§^
MMSE (/30), mean ± SD			
All participants	21.4 ± 5.5	20.3 ± 6.1	0.200^‡^
Preserved WP group	23.3 ± 4.7	21.5 ± 4.6	0.059^‡^
Reduced WP group	18.8 ± 5.5	17.7 ± 7.9	0.515^‡^
FAB (/18), mean ± SD			
All participants	10.1 ± 3.5 (n = 85)	9.5 ± 3.7	0.327^‡^
Preserved WP group	11.3 ± 2.8 (n = 50)	10.1 ± 3.4	0.052^‡^
Reduced WP group	8.3 ± 3.7 (n = 35)	8.3 ± 4.0	0.997^‡^
TMT-A (s), mean ± SD			
All participants	151 ± 119 (n = 80)	132 ± 101 (n = 63)	0.289^‡^
Preserved WP group	118 ± 93 (n = 50)	118 ± 85 (n = 44)	0.983^‡^
Reduced WP group	207 ± 137 (n = 30)	164 ± 126 (n = 19)	0.271^‡^
TUG (s), mean ± SD			
All participants	24.1 ± 30.6 (n = 83)	24.1 ± 30.0 (n = 65)	0.994^‡^
Preserved WP group	14.7 ± 5.0	13.9 ± 4.6	0.408^‡^
Reduced WP group	39.2 ± 45.4 (n = 32)	53.0 ± 48.3 (n = 17)	0.328^‡^
Hypertension			
All participants	43 (49.4%)	47 (66.2%)	0.034^†^
Preserved WP group	23 (45.1%)	36 (75.0%)	0.002^†^
Reduced WP group	20 (55.6%)	11 (47.8%)	0.562^†^
Diabetes mellitus			
All participants	18 (20.7%)	22 (31.0%)	0.139^†^
Preserved WP group	10 (19.6%)	17 (35.4%)	0.078^†^
Reduced WP group	8 (22.2%)	5 (21.7%)	0.965^†^
Hyperlipidaemia			
All participants	33 (37.9%)	32 (45.1%)	0.364^†^
Preserved WP group	19 (37.3%)	25 (52.1%)	0.138^†^
Reduced WP group	14 (38.9%)	7 (30.4%)	0.508^†^
Smoking			
All participants	27 (31.0%)	20 (28.2%)	0.695^†^
Preserved WP group	20 (39.2%)	15 (31.3%)	0.407^†^
Reduced WP group	7 (19.4%)	5 (21.7%)	0.831^†^
Ischaemic stroke			
All participants	31 (41.9%) (n = 74)	33 (54.1%) (n = 61)	0.157^†^
Preserved WP group	21 (46.7%) (n = 45)	21 (47.7%) (n = 44)	0.920^†^
Reduced WP group	10 (34.5%) (n = 29)	12 (70.6%) (n = 17)	0.018^†^
Shunt procedures (VP/LP)			
All participants	27/60	24/47	0.711^†^
Preserved WP group	17/34	17/31	0.827^†^
Reduced WP group	10/26	7/16	0.826^†^

*FAB* Frontal Assessment Battery, *iNPH* idiopathic normal-pressure hydrocephalus, *iNPHGS* idiopathic Normal-Pressure Hydrocephalus Grading Scale, *LP* lumboperitoneal, *MMSE* Mini-Mental State Examination, *TMT-A* Trail Making Test A, *SINPHONI-2* a multicentre prospective trial of lumboperitoneal shunt surgery for patients with idiopathic normal-pressure hydrocephalus, *TUG* timed up-and-go test, *VP* ventriculoperitoneal, *WP* walking performance.

^†^The chi-square test was used.

^‡^Student’s t-test was used.

^§^The Mann–Whitney U-test was used.

The prevalences of hypertension and ischaemic stroke history in patients with each mRS score are shown in Fig. 2-b. Among the patients with mRS grade 1 or 2, those with unfavourable outcomes had a significantly higher prevalence of hypertension than those with favourable outcomes (p < 0.001). Among the patients with mRS grade 3, the prevalence of hypertension in patients with unfavourable outcomes tended to be higher than that in patients with favourable outcomes (p = 0.057). By contrast, among the patients with mRS score 4, the prevalence of hypertension in patients with unfavourable outcomes was significantly lower than that in those with favourable outcomes (p = 0.003). Among the patients with each mRS score, there was no significant difference in the prevalence of ischaemic stroke history between patients with favourable and unfavourable outcomes (mRS grade 1 or 2, p = 0.514; mRS grade 3, p = 0.589; and mRS grade 4, p = 0.100).

### Changes in clinical measures after shunt implantation

The changes in clinical measures after shunt implantation are shown in Table 3 (all participants and each working performance group) and Supplementary Table 2 (each sample). The analyses targeting all participants demonstrated that the participants with favourable outcomes showed significant improvements in iNPHGS sub- and total scores, the scores of all neuropsychological tests, and TUG completion time, whereas participants with unfavourable outcomes showed significant improvements only in iNPH gait, urination sub- and total scores, and TUG completion time. In the preserved walking performance group, patients with favourable outcomes showed significant improvements in mRS score, iNPHGS sub- and total scores, TUG completion time, and scores of all neuropsychological tests except for TMT-A, whereas patients with unfavourable outcomes did not show significant improvement in any clinical measures we administered except for TUG completion time and iNPHGS total scores. In the reduced walking performance group, patients with favourable outcomes showed significant improvements in mRS score, iNPHGS sub- and total scores, TUG completion time, and the scores of all neuropsychological tests except for MMSE, whereas patients with unfavourable outcomes showed significant improvement only in iNPHGS gait sub- and total scores and TUG completion time.

**Table 3:** Changes in clinical measures after shunt implantation.

Variable	Patients with favourable outcomes	p value	Patients with unfavourable outcomes	p value
**Number**				
All participants	87 (55.1%)		71 (44.9%)	
Preserved WP group	51 (51.5%)		48 (48.5%)	
Reduced WP group	36 (61.0%)		23 (39.0%)	
**mRS, median (25th–75th percentile)**				
All participants	− 1.0 (− 1.0 to 1.0)	< 0.001^†^	(0.0 to 0.0)	0.002^†^
Preserved WP group	− 1.0 (− 1.0 to 1.0)	< 0.001^†^	0.0 (0.0 to 0.0)	0.006^†^
Reduced WP group	− 1.0 (− 1.5 to 1.0)	< 0.001^†^	0.0 (0.0 to 0.0)	0.157^†^
**iNPHGS, median (25th–75th percentile)**				
Gait				
All participants	− 1.0 (− 2.0 to 0.0)	< 0.001^†^	(− 1.0 to 0.0) (n = 70)	0.013^†^
Preserved WP group	− 1.0 (− 1.0 to 0.0)	< 0.001^†^	(− 1.0 to 0.0) (n = 47)	0.102^†^
Reduced WP group	− 1.0 (− 2.0 to 1.0)	< 0.001^†^	0.0 (− 1.0 to 0.0)	0.014^†^
Cognition				
All participants	− 1.0 (− 1.0 to 0.0)	< 0.001^†^	(0.0–0.0) (n = 70)	0.577^†^
Preserved WP group	− 1.0 (− 1.0 to 0.0)	< 0.001^†^	(0.0–0.0) (n = 47)	0.513^†^
Reduced WP group	− 1.0 (− 1.0 to 0.0)	< 0.001^†^	0.0 (0.0–0.0)	1.000^†^
Urination				
All participants	− 1.0 (− 1.0 to 0.0)	< 0.001^†^	(− 1.0 to 0.0) (n = 70)	0.013^†^
Preserved WP group	− 1.0 (− 1.0 to 0.0)	< 0.001^†^	(− 1.0 to 0.0) (n = 47)	0.082^†^
Reduced WP group	− 1.0 (− 2.0 to 0.0)	< 0.001^†^	0.0 (− 1.0 to 0.0)	0.075^†^
Total				
All participants	− 3.0 (− 4.0 to 2.0)	< 0.001^†^	(− 1.0 to 0.0) (n = 70)	0.004^†^
Preserved WP group	− 2.0 (− 4.0 to 1.0)	< 0.001^†^	(− 1.0 to 0.0) (n = 47)	0.041^†^
Reduced WP group	− 3.0 (− 4.0 to 2.0)	< 0.001^†^	0.0 (− 2.0 to 0.0)	0.036^†^
MMSE, mean ± SD				
All participants	± 3.5	0.003^‡^	0.2 ± 3.7	0.675^‡^
Preserved WP group	± 3.1	0.019^‡^	− 0.1 ± 2.7	0.710^‡^
Reduced WP group	1.3 ± 4.0	0.063^‡^	0.9 ± 5.1	0.426^‡^
FAB, mean ± SD				
All participants	± 2.6 (n = 84)	< 0.001^‡^	0.6 ± 2.6	0.053^‡^
Preserved WP group	± 2.3 (n = 50)	< 0.001^‡^	0.4 ± 2.3	0.234^‡^
Reduced WP group	1.5 ± 3.0 (n = 34)	0.006^‡^	1.0 ± 3.2	0.127^‡^
TMT− A (s), mean ± SD				
All participants	− 25 ± 68 (n = 79)	0.002^‡^	− 3 ± 66 (n = 44)	0.736^‡^
Preserved WP group	− 18 ± 63 (n = 50)	0.051^‡^	− 3 ± 40 (n = 44)	0.602^‡^
Reduced WP group	− 36 ± 76 (n = 29)	0.015^‡^	− 2 ± 105 (n = 19)	0.937^‡^
TUG (s), mean ± SD				
All participants	− 8.6 ± 26.2 (n = 83)	0.004^‡^	− 5.4 ± 12.3	< 0.001^‡^
Preserved WP group	− 3.4 ± 3.0	< 0.001^‡^	− 1.9 ± 3.4	< 0.001^‡^
Reduced WP group	− 17.0 ± 41.1 (n = 32)	0.026^‡^	− 15.6 ± 20.5 (n = 17)	0.006^‡^

*FAB* Frontal Assessment Battery, *iNPH* idiopathic normal-pressure hydrocephalus, *iNPHGS* idiopathic Normal-Pressure Hydrocephalus Grading Scale, *MMSE* Mini-Mental State Examination, *mRS* modified Rankin Scale, *TMT-A* Trail Making Test A, *TUG* timed up-and-go test, *WP* walking performance.

^†^The Wilcoxon signed-ranked test was used.

^‡^The paired Student’s t-test was used.

### Predictors of unfavourable outcomes after shunt surgery

The results of multivariate logistic analyses are shown in Table 4. Multicollinearity was not found in any of the analyses. For the analysis targeting all participants, disease duration, the iNPHGS urination subscore, the prevalence of hypertension, and Evans’ index were selected. All of these variables were significant risk factors for unfavourable outcomes after shunt surgery. In the preserved walking performance group, disease duration, total MMSE score (VIF = 1.816), FAB score (VIF = 1.877), and the prevalence of hypertension and diabetes mellitus (VIF = 1.099) were selected. Among these variables, disease duration and hypertension were significant risk factors for unfavourable outcomes after shunt surgery. In the reduced walking performance group, the iNPHGS urination subscore (VIF = 1.027), the prevalence of ischaemic stroke history (VIF = 1.059), and Evans’ index were selected for the analysis. Of these variables, Evans’ index was the only significant risk factor for unfavourable outcomes after shunt surgery.

**Table 4:** Results of multivariate logistic regression analyses.

Variable	Odds ratio	95% CI	VIF	p value
**A. All participants**				
Disease duration	1.25 (calculated for 1-year increment)	1.05–1.48	1.034	0.012
iNPHGS urination subscore	1.48 (calculated for 1 increment)	1.05–2.08	1.013	0.024
Hypertension	2.16	1.07–4.40	1.025	0.033
Evans’ index	0.80 (calculated for 0.01 increment)	0.70–0.92	1.046	0.002
**B. Preserved walking performance group**				
Disease duration	1.25 (calculated for 1-year increment)	1.01–1.56	1.052	0.045
Hypertension	4.23	1.72–10.4	1.113	0.002
**C. Reduced walking performance group**				
Evans’ index	0.71 (calculated for 0.01 increment)	0.55–0.90	1.052	0.045

*CI* confidence interval, *iNPHGS* idiopathic Normal-Pressure Hydrocephalus Grading Scale, *VIF* variance inflation factor.

## Discussion

### Predictors of unfavourable outcomes in iNPH patients

In the analyses targeting all participants, we showed that the presence of hypertension, more severe urinary dysfunction evaluated by the iNPHGS urination subscore, relatively smaller lateral ventricles for hydrocephalus measured using Evans’ index, and longer disease duration were predictors of unfavourable outcomes after shunt surgery. In our results, hypertension was the only VRF that could predict shunt responsiveness. In the subgroup analyses, hypertension was more associated with iNPH patients with independent walking than those who needed assistance to walk or could not walk.

A few studies have reported whether VRFs are associated with outcomes after shunt surgery^6,9–11^, but their results are inconsistent. Boon et al. demonstrated that the proportion of shunt responders among iNPH patients with CVD was significantly lower than that among iNPH patients without CVD^6^. Andrén et al. reported that the presence of vascular comorbidity had almost no negative impact on the outcome 2–6 years after shunt surgery, but patients with hypertension and/or a history of stroke showed a less favourable progression on the self-assessed mRS only 6 years after shunt surgery^10^. In the study by Klinge et al., the presence of VRFs was not correlated with the magnitude of improvement after shunt surgery^11^. Taking mRS score as a measure of physical disability, including gait disturbance, the patients’ severity in Boon et al.’s study was near that among patients in our study who needed assistance to walk or could not walk, and the patients’ severity in Andrén et al.’s study was almost equivalent to that of patients in our study with independent walking. As with our study, Boon et al.’s and Andrén et al.’s studies revealed an association between VRFs and poor outcomes after shunt surgery. However, our study did not reveal any association between hypertension and long-term outcomes, and Andrén et al. did not find any association between hypertension and short-term outcomes.

In addition, the subgroup analyses of patients with independent walking revealed that the MMSE and FAB total scores in patients with unfavourable outcomes tended to be lower than in those with favourable outcomes, although these findings were not statistically significant. These results were coincident with the expectations derived from our hypothesis that hypertension might be more closely related to AD than to iNPH pathology. However, Nakajima et al. reported that patients with mRS grade 3 at baseline showed an association between comorbid AD and unfavourable outcomes^9^. Of these patients, those who had unfavourable outcomes had a significantly lower prevalence of hypertension than those who had favourable outcomes. These findings are incompatible with our results and hypothesis, even though the diagnostic process for comorbid AD used by Nakajima et al. was uncertain. Interestingly, we observed the same improvement effect of hypertension on iNPH symptoms after shunt surgery that Nakajima et al. did, although only in patients with mRS grade 4 at baseline in our study. Hypertension seems to play a bidirectional role in shunt responsiveness, so the effect of hypertension on shunt responsiveness may be offset, depending on the severity of physical disability in patients with iNPH. Although the mRS is a well-validated and reliable tool for evaluating activities of daily life or physical disability, interobserver studies closest in design to large-scale clinical trials demonstrate potentially significant interobserver variability^26^. Large-scale cohort studies with improved evaluations of activities of daily life and/or physical disability are needed to explain these discrepancies in results.

Previous studies have reported that hypertension is one of the common risk factors for the pathogenesis of iNPH^5–8^. However, the prevalence of hypertension in our patients was lower than that in septuagenarian men (74.7–81.4%) and women (69.2–72.3%) between 2010 and 2016 in Japan, except among patients with independent walking who had unfavourable outcomes after shunt surgery^27^. Previous studies have also indicated a lower prevalence of hypertension in patients with iNPH, except in Israelsson et al.’s study^6,8–11^. It should be reconsidered whether hypertension is actually related to the pathogenesis of iNPH. The glymphatic and intramural periarterial drainage pathways, which play a crucial role in maintaining brain interstitial fluid homeostasis and washing out waste products, have recently drawn attention not only in AD but also in iNPH studies^28–32^. It is thought that these pathways are driven by pulsations of the arterial wall^33^. A recent rat study revealed that hypertension increases CSF backflow and thereby reduces CSF net flow in the perivascular space^34^. Although impairments in these pathways related to hypertension may be associated not only with the decline in amyloid beta clearance from the central nervous system but also with ventriculomegaly in iNPH, our and Nakajima et al.’s results suggest the possibility that comorbid hypertension facilitates the drainage of CSF through an inserted catheter or through the perivascular space after shunt surgery in some iNPH patients.

In the present study, more severe urinary dysfunction was also associated with unfavourable outcomes after shunt surgery in patients with iNPH. To the best of our knowledge, no previous studies have demonstrated an association between urinary dysfunction and shunt responsiveness in patients with iNPH. It is known that patients with iNPH have urge and functional urinary incontinence^35^. However, urinary dysfunction is common in elderly individuals, and urge and functional urinary incontinence are also observed in patients with other neurological diseases, such as cerebrovascular disease, AD, or Parkinson’s disease^36^. Therefore, there was a possibility that some iNPH patients with severe urinary dysfunction had comorbid neurological disease, which led to unfavourable outcomes after shunt surgery in the iNPH patients.

A longer disease duration was also related to unfavourable outcomes after shunt surgery in patients with iNPH. This finding is consistent with previous studies. Kimura et al. and Vakili et al. reported that a longer symptom duration was associated with less improvement in mRS at 6 months or 1 year postoperatively^37,38^. In addition, in our study, a longer disease duration was more associated with iNPH patients with independent walking than with those who needed assistance to walk or could not walk. We speculate that this discrepancy between the two groups might arise from some comorbid neurological disease in some patients with independent walking, such as AD, which does not result in severe gait disturbance in the early and moderate stages of the disease. If these patients might have asymptomatic ventriculomegaly with features of iNPH on MRI, their symptoms might be not mainly due to iNPH but due to comorbid neurological diseases^2^.

Having fairly small lateral ventricles at baseline was also associated with an unfavourable outcome after shunt surgery. Moreover, in the subgroup analyses, this finding was more associated with iNPH patients who needed assistance to walk or could not walk than those with independent walking. To the best of our knowledge, there has been no report of a significant association between ventricular size and symptom severity at baseline or between ventricular size at baseline and shunt responsiveness in patients with iNPH. However, several previous studies have reported that a reduction in ventricular size after shunt implantation was positively associated with the magnitude of clinical improvement in patients with iNPH^39,40^. The relatively small lateral ventricles in hydrocephalus at baseline may reflect low elasticity and plasticity of the brain parenchyma. However, a significant association between ventricular size at baseline and shunt responsiveness was not observed in the patients with independent walking. One possible reason is that ventricular dilation was caused not only by iNPH-related pathology but also by AD- and/or CVD-related pathology. Because of these conflicting factors influencing reversibility after shunt surgery, ventricular size at baseline could not predict shunt responsiveness in patients with independent walking.

### Participants from Tohoku University Hospital and SINPHONI-2

Among the participants from Tohoku University Hospital, more could walk independently and fewer needed assistance to walk or could not walk than among the participants from SINPHONI-2. In addition, patients from Tohoku University showed shorter TUG completion times than those from SINPHONI-2. Although the combination of the two samples allowed us to investigate iNPH patients with milder to more severe gait disturbances, it should be noted that the results in patients with independent walking might have been easily influenced by the measurement bias related to the single-hospital nature of the data. Moreover, the disease duration of the participants from Tohoku University Hospital was significantly longer than that from SINPHONI-2. Although these findings might be true, it might be that the evaluators in Tohoku University Hospital tended to take a more detailed medical history than those in the other institutes that participated in SINPHONI-2.

The participants from Tohoku University Hospital were less likely to have favourable outcomes than those from SINPHONI-2, although this finding was not statistically significant. The quality of shunt surgery seemed not to be associated with poorer outcomes of shunt surgery in Tohoku University Hospital because the number of serious adverse events was lower in the participants from Tohoku University Hospital than in the participants from SINPHONI-2. One possible reason for the poorer outcomes in the Tohoku University Hospital cohort is that these participants had a longer disease duration than those from SINPHONI-2^37,38^. Another possible reason is that evaluators at Tohoku University Hospital could have tended to overestimate symptom severity and/or underestimate the improvements in symptoms after shunt surgery when evaluated using severity scales. Among participants with independent walking, the mean TUG completion time in those from Tohoku University Hospital was significantly shorter than that in SINPHONI-2 patients, whereas the median iNPHGS gait subscore in participants from Tohoku University Hospital tended to be greater. In addition, the improvements in cognitive deficits as assessed by the MMSE and FAB in participants from Tohoku University Hospital were more marked than they were in the SINPHONI-2 groups, even though the magnitude of improvements in cognitive deficits as measured by the iNPHGS cognition subscore was equivalent between the two groups (Supplementary Table 2).

### Limitation

The present study has several limitations. First, we did not evaluate CSF biomarkers for AD, perform amyloid imaging, or test memory function. Therefore, we cannot state with certainty that hypertension was associated with the presence of AD pathology in patients with independent walking. Second, the results in patients with independent walking might have been influenced by measurement bias because almost 60% of these patients were recruited from Tohoku University Hospital. Third, we were able to check the presence of ischaemic lesions on presurgical MRI only for approximately 70% of the participants from SINPHONI-2 but for all the participants from Tohoku University Hospital. Therefore, the prevalence of a history of ischaemic stroke was more influenced by the trends in the participants from Tohoku University Hospital.

## Conclusions

Our study demonstrated that hypertension was the only VRF that could predict unfavourable outcomes after shunt surgery in patients with iNPH. In addition, more severe urinary dysfunction, relatively smaller lateral ventricles in hydrocephalus, and longer disease duration were also predictors. Larger-scale studies of comorbidities, including AD and CSF dynamics, are needed to elucidate the reason why hypertension is related to the irreversibility of symptoms after shunt placement.

## Supplementary Information

Supplementary Tables.

## Data Availability

The datasets used and/or analysed during the current study are not publicly available due to participant’s privacy protections but are available from the corresponding author upon reasonable request.

## References

[1] Adams RD, Fisher CM, Hakim S, Ojemann RG, Sweet WH (1965). Symptomatic occult hydrocephalus with “normal” cerebrospinal-fluid pressure. A treatable syndrome. N. Engl. J. Med..

[2] Iseki C (2009). Asymptomatic ventriculomegaly with features of idiopathic normal pressure hydrocephalus on MRI (AVIM) in the elderly: A prospective study in a Japanese population. J. Neurol Sci..

[3] Hashimoto M, Ishikawa M, Mori E, Kuwana N (2010). Diagnosis of idiopathic normal pressure hydrocephalus is supported by MRI-based scheme: a prospective cohort study. Cerebrospinal Fluid Res..

[4] Kazui H, Miyajima M, Mori E, Ishikawa M, SINPHONI-2 Investigators (2015). Lumboperitoneal shunt surgery for idiopathic normal pressure hydrocephalus (SINPHONI-2): An open-label randomised trial. Lancet Neurol..

[5] Graff-Radford NR, Godersky JC (1987). Idiopathic normal pressure hydrocephalus and systemic hypertension. Neurology.

[6] Boon AJ (1999). Dutch normal-pressure hydrocephalus study: The role of cerebrovascular disease. J. Neurosurg..

[7] Malm J (2013). Influence of comorbidities in idiopathic normal pressure hydrocephalus—Research and clinical care. A report of the ISHCSF task force on comorbidities in INPH. Fluids Barriers CNS..

[8] Israelsson H, Larsson J, Eklund A, Malm J (2020). Risk factors, comorbidities, quality of life, and complications after surgery in idiopathic normal pressure hydrocephalus: Review of the INPH-CRasH study. Neurosurg. Focus.

[9] Nakajima M (2019). Background risk factors associated with shunt intervention for possible idiopathic normal pressure hydrocephalus: A Nationwide Hospital-Based Survey in Japan. J. Alzheimers Dis..

[10] Andrén K (2018). Long-term effects of complications and vascular comorbidity in idiopathic normal pressure hydrocephalus: A quality registry study. J. Neurol..

[11] Klinge P, Hellström P, Tans J, Wikkelsø C, European iNPH Multicentre Study Group (2012). One-year outcome in the European multicentre study on iNPH. Acta Neurol. Scand..

[12] Breteler MM (2000). Vascular risk factors for Alzheimer's disease: An epidemiologic perspective. Neurobiol. Aging.

[13] de la Torre JC (2012). Cerebral hemodynamics and vascular risk factors: Setting the stage for Alzheimer's disease. J. Alzheimers Dis..

[14] Daugherty AM (2021). Hypertension-related risk for dementia: A summary review with future directions. Semin. Cell Dev. Biol..

[15] Hamilton R (2010). Lack of shunt response in suspected idiopathic normal pressure hydrocephalus with Alzheimer disease pathology. Ann. Neurol..

[16] Leinonen V (2012). Cortical brain biopsy in long-term prognostication of 468 patients with possible normal pressure hydrocephalus. Neurodegener. Dis..

[17] Kazui H (2016). Association between high biomarker probability of Alzheimer's disease and improvement of clinical outcomes after shunt surgery in patients with idiopathic normal pressure hydrocephalus. J. Neurol. Sci..

[18] Day GS (2017). Differentiating cognitive impairment due to corticobasal degeneration and Alzheimer disease. Neurology.

[19] Bådagård H, Braun M, Nilsson D, Stridh L, Virhammar J (2020). Negative predictors of shunt surgery outcome in normal pressure hydrocephalus. Acta Neurol. Scand..

[20] van Swieten JC, Koudstaal PJ, Visser MC, Schouten HJ, van Gijn J (1988). Interobserver agreement for the assessment of handicap in stroke patients. Stroke.

[21] Kubo Y (2008). Validation of grading scale for evaluating symptoms of idiopathic normal-pressure hydrocephalus. Dement. Geriatr. Cogn. Disord..

[22] Podsiadlo D, Richardson S (1991). The timed ‘‘Up & Go’’: A test of basic functional mobility for frail elderly persons. J. Am. Geriatr. Soc..

[23] Folstein MF, Folstein SE, McHugh PR (1975). ‘‘Mini-mental state’’. A practical method for grading the cognitive state of patients for the clinician. J. Psychiatr. Res..

[24] Dubois B, Slachevsky A, Litvan I, Pillon B (2010). The FAB: A frontal assessment battery at bedside. Neurology.

[25] Reitan R (1958). Validity of the trail making test as an indicator of organic brain damage. Percept. Mot. Skills.

[26] Quinn TJ, Dawson J, Walters MR, Lees KR (2009). Reliability of the modified Rankin Scale: A systematic review. Stroke.

[27] Hisamatsu, T. & Miura, K. Epidemiology and control of hypertension in Japan: A comparison with Western countries. *J. Hum. Hypertens.*10.1038/s41371-021-00534-3 (2021). Online ahead of print. 10.1038/s41371-021-00534-3 33854177

[28] Iliff JJ (2012). A paravascular pathway facilitates CSF flow through the brain parenchyma and the clearance of interstitial solutes, including amyloid beta. Sci. Transl. Med..

[29] Carare RO, Hawkes CA, Weller RO (2014). Afferent and efferent immunological pathways of the brain. Anatomy, function and failure. Brain Behav. Immun..

[30] Weller RO, Djuanda E, Yow HY, Carare RO (2009). Lymphatic drainage of the brain and the pathophysiology of neurological disease. Acta Neuropathol..

[31] Reeves BC (2020). Glymphatic system impairment in Alzheimer's disease and idiopathic normal pressure hydrocephalus. Trends Mol. Med..

[32] Ringstad G, Vatnehol SAS, Eide PK (2017). Glymphatic MRI in idiopathic normal pressure hydrocephalus. Brain.

[33] Iliff JJ (2013). Cerebral arterial pulsation drives paravascular CSF-interstitial fluid exchange in the murine brain. J. Neurosci..

[34] Mestre H (2018). Flow of cerebrospinal fluid is driven by arterial pulsations and is reduced in hypertension. Nat. Commun..

[35] Sakakibara R (2012). Correlation of right frontal hypoperfusion and urinary dysfunction in iNPH: A SPECT study. Neurourol. Urodyn..

[36] Erdem K, Chu FM (2006). Management of overactive bladder and urge urinary incontinence in the elderly patient. Am J Med..

[37] Kimura T (2021). Preoperative predictive factors of short-term outcome in idiopathic normal pressure hydrocephalus. World Neurosurg..

[38] Vakili S (2016). Timing of surgical treatment for idiopathic normal pressure hydrocephalus: Association between treatment delay and reduced short-term benefit. Neurosurg. Focus..

[39] Neikter J (2020). Ventricular volume is more strongly associated with clinical improvement than the Evans index after shunting in idiopathic normal pressure hydrocephalus. AJNR. Am. J. Neuroradiol..

[40] Hiraoka K (2010). Changes in the volumes of the brain and cerebrospinal fluid spaces after shunt surgery in idiopathic normal-pressure hydrocephalus. J. Neurol. Sci..

