# Supplementary material for: Risk factors for unfavourable outcomes after shunt surgery in patients with idiopathic normal-pressure hydrocephalus
Source: Sci Rep. 2022 Aug 17;12:13921. doi: 10.1038/s41598-022-18209-5 (PMC9385629; doi:10.1038/s41598-022-18209-5)
Supplement: Supplementary file 1 — Supplementary Tables. [file 41598_2022_18209_MOESM1_ESM.pdf]

Supplementary Table 1. Adverse events during the 1-year follow-up period in the participants from the Tohoku University Hospital.

A. Serious adverse events

Event	Number of events
Lumbar radicular pain requiring surgical repair	3
Inappropriate placement of ventricular catheter requiring revision	1
Cerebral infarction	1
Myocardial infarction	1
Mandibular fracture	1
Delusional disorder	1
Pneumonia	1
Total	9 events in 9 patients (11.5%)

B. Non-serious adverse events

Event	Number of events
Postural headache	16
Subdural haematoma not requiring surgery	5
Lumber compression fracture	4
Asymptomatic intracerebral haemorrhage immediately after shunt insertion not requiring surgery	4

Infection	*3
Forearm fracture	2
Asymptomatic subdural effusion	1
Deep vein thrombosis	1
Duodenal ulcer	1
Shoulder fracture	1
Total	38 events in 31 patients (39.7%)

*Two patients had urinary tract infection, and the remaining patient had catheter-related bloodstream infection.

Supplementary Table 2. Changes in clinical measures after shunt implantation in each sample.

A. Tohoku University Hospital

Variables		Patients with favorable outcomes	p-value	Patients with unfavorable outcomes	p-value
Number	All participants	37 (47.4%)		41 (52.6%)	
	- Preserved WP group	26 (45.6%)		31 (54.4%)	
	- Reduced WP group	11 (52.4%)		10 (47.6%)	
mRS, median (25th–75th percentile)	All participants	-1.0 (-1.0--1.0)	<0.001†	0.0 (0.0–0.0)	0.020†
	- Preserved WP group	-1.0 (-1.0--1.0)	<0.001†	0.0 (0.0–0.0)	0.034†
	- Reduced WP group	-1.0 (-1.0--1.0)	0.002†	0.0 (0.0–0.0)	0.317†
iNPHGS, median (25–75th percentile)					

Gait	All participants	-1.0 (-2.0--1.0)	<0.001†	0.0 (0.0–0.0) (n = 40)	0.005†
	- Preserved WP group	-1.0 (-2.0--1.0)	<0.001†	0.0 (0.0–0.0) (n = 30)	0.014†
	- Reduced WP group	-1.0 (-2.0--1.0)	0.004†	0.0 (0.0–0.0)	0.157†
Cognition	All participants	-1.0 (-1.0–0.0)	<0.001†	0.0 (0.0–0.0) (n = 40)	0.052†
	- Preserved WP group	-1.0 (-1.0–0.0)	<0.001†	0.0 (0.0–0.0) (n = 30)	0.058†
	- Reduced WP group	-1.0 (-1.0–0.0)	0.007††	0.0 (0.0–0.0)	0.564†
Urination	All participants	-1.0 (-1.0–0.0)	<0.001†	0.0 (-1.0–0.0) (n = 40)	0.040†
	- Preserved WP group	-1.0 (-1.0–0.0)	0.001†	0.0 (-1.0–0.0) (n = 30)	0.134†
	- Reduced WP group	-1.0 (-1.0–0.0)	0.070†	0.0 (0.0–0.0)	0.180†
Total	All participants	-2.0 (-3.0--2.0)	<0.001†	0.0 (-1.0–0.0) (n = 40)	0.002†
	- Preserved WP group	-2.0 (-3.0--1.0)	<0.001†	0.0 (-1.0–0.0) (n = 30)	0.010†
	- Reduced WP group	-2.0 (-3.0--2.0)	0.003†	0.0 (-2.0–1.0)	0.102†

MMSE (/30), mean ± SD	All participants	2.4 ± 3.8	<0.001‡	0.1 ± 2.8	0.825‡
	- Preserved WP group	2.0 ± 3.3	0.005‡	0.3 ± 2.6	0.578‡
	- Reduced WP group	3.2 ± 4.9	0.056‡	-0.4 ± 3.6	0.733‡
FAB (/18), mean ± SD	All participants	2.3 ± 2.6	<0.001‡	0.5 ± 2.7	0.224‡
	- Preserved WP group	1.8 ± 2.8	0.003‡	0.4 ± 2.4	0.342‡
	- Reduced WP group	3.6 ± 1.4 (n = 10)	<0.001‡	0.8 ± 3.4	0.479‡
TMT-A (seconds), mean ± SD	All participants	-40 ± 82	0.006‡	-8 ± 75	0.483‡
	- Preserved WP group	-27 ± 77	0.087‡	-4 ± 43	0.595‡
	- Reduced WP group	-75 ± 91 (n = 10)	0.029‡	-21 ± 135	0.637‡
TUG (seconds), mean ± SD	All participants	-4.6 ± 6.3	<0.001‡	-4.8 ± 3.4	0.048‡
	- Preserved WP group	-3.5 ± 2.8	<0.001‡	-1.9 ± 3.4	0.003‡
	- Reduced WP group	-2.5 (-11.1--2.5) (n = 7)	0.018[a]	-11.4 (-50.0--0.3) (n = 5)	0.080[a]

B. SINPHONI-2

Variables		Patients with favorable outcomes	p-value	Patients with unfavorable outcomes	p-value
Number	All participants	50 (62.5%)		30 (37.5%)	
	- Preserved WP group	25 (59.5%)		17 (40.5%)	
	- Reduced WP group	25 (65.8%)		13 (34.2%)	
mRS, median (25th–75th percentile)	All participants	-1.0 (-2.0--1.0)	<0.001†	0.0 (0.0–0.0)	0.038†
	- Preserved WP group	-1.0 (-1.5--1.0)	<0.001†	0.0 (0.0–0.5)	0.063†
	- Reduced WP group	-1.0 (-2.0--1.0)	<0.001†	0.0 (0.0–0.0)	0.317†
iNPHGS, median (25th–75th percentile)					
Gait	All participants	-1.0 (-1.0–0.0)	<0.001†	0.0 (-1.0–0.0)	0.310†

	- Preserved WP group	-1.0 (-1.0–0.0)	<0.001†	0.0 (-1.0–0.0)	0.831†
	- Reduced WP group	-1.0 (-1.5--0.5)	<0.001†	0.0 (-1.0–0.0)	0.046†
Cognition	All participants	-1.0 (-1.0–0.0)	<0.001†	0.0 (0.0–1.0)	0.317†
	- Preserved WP group	-1.0 (-1.0–0.0)	<0.001†	0.0 (-0.5–1.0)	0.366†
	- Reduced WP group	-1.0 (-1.0–0.0)	<0.001†	0.0 (0.0–0.5)	0.655†
Urination	All participants	-1.0 (-2.0–0.0)	<0.001†	0.0 (-1.0–0.0)	0.151†
	- Preserved WP group	-1.0 (-2.0–0.0)	<0.001†	0.0 (-1.0–0.0)	0.366†
	- Reduced WP group	-1.0 (-2.0–0.0)	0.001†	0.0 (-1.0–0.5)	0.271†
Total	All participants	-3.0 (-4.0--2.0)	<0.001†	0.0 (-1.0–0.0)	0.335†
	- Preserved WP group	-2.0 (-4.0--1.0)	<0.001†	0.0 (-1.5–1.0)	0.812†
	- Reduced WP group	-3.0 (-4.0--2.0)	<0.001†	0.0 (-1.5–0.0)	0.176†
MMSE, mean ± SD	All participants	0.3 ± 3.0	0.538‡	0.3 ± 4.6	0.726‡

	- Preserved WP group	0.1 ± 2.6	0.880‡	-0.9 ± 2.9	0.223‡
	- Reduced WP group	0.4 ± 3.3	0.512‡	1.8 ± 6.0	0.292‡
FAB, mean ± SD	All participants	0.6 ± 2.4 (n = 48)	0.077‡	0.7 ± 2.5	0.123‡
	- Preserved WP group	0.6 ± 1.6 (n = 24)	0.061‡	0.4 ± 2.1	0.490‡
	- Reduced WP group	0.6 ± 3.0 (n = 24)	0.326‡	1.2 ± 3.1	0.173‡
TMT-A (seconds), mean ± SD	All participants	-12 ± 51 (n = 43)	0.139‡	7 ± 45	0.448‡
	- Preserved WP group	-8 ± 43 (n = 24)	0.370‡	-1 ± 33	0.930‡
	- Reduced WP group	-16 ± 60 (n = 19)	0.253‡	19 ± 57 (n = 9)	0.343‡
TUG (seconds), mean ± SD	All participants	-11.3 ± 33.3	0.020‡	-6.3 ± 9.9	0.002‡
	- Preserved WP group	-3.2 ± 3.3	<0.001‡	-1.7 ± 3.5	0.061‡
	- Reduced WP group	-19.4 ± 46.0	0.046‡	0.046‡	0.005‡

†The Wilcoxon signed-ranked test was used. ‡A paired Student's t-test was used. [a] The Wilcoxon signed-ranked test was used because of small number of samples.

FAB: the frontal assessment battery; iNPH: idiopathic normal pressure hydrocephalus; iNPHGS: the idiopathic Normal Pressure Hydrocephalus Grading Scale; MMSE: the Mini-Mental State Examination; mRS: the modified Rankin Scale; SINPHONI-2: a multicenter prospective trial of lumboperitoneal shunt surgery for patients with idiopathic normal pressure hydrocephalus; TMT-A: the Trail Making test A; TUG: the Timed Up and Go Test; WP: walking performance.
